# Not Your Typical Cyst: A Periduodenal Chylous Cyst Diagnosed via Endoscopic Ultrasound

**DOI:** 10.7759/cureus.27284

**Published:** 2022-07-26

**Authors:** Chiemeziem Eke, Ian Greenberg, Prashant Kedia

**Affiliations:** 1 Gastroenterology, Methodist Dallas Medical Center, Dallas, USA

**Keywords:** fine needle aspiration, retroperitoneal, mesenteric cyst, endoscopic ultrasound, duodenum

## Abstract

Chylous mesenteric cysts represent a subgroup of uncommon, mostly benign intra-abdominal masses often identified incidentally on radiographs. Diagnosis is often challenging because these lesions cannot be reliably distinguished from hematomas or other cystic lesions without direct tissue sampling. In addition, data remains scarce regarding nonsurgical diagnostic and therapeutic options for these cysts. This case describes an endoscopic option for a uniquely located chylous cyst.

## Introduction

Mesenteric cysts are a rare GI condition of unclear etiology thought to affect those of all ages [1]. These cysts are typically asymptomatic and are usually discovered by chance. Ultrasound and CT can aid in diagnosis and therapy [2]. The mainstay of therapy has been surgical intervention, particularly for large symptomatic cysts [1-5]. To date, the literature describing the endosonographic diagnosis of such cysts is limited. Presently, no recommendations exist to tailor endoscopic management of mesenteric cysts.

## Case presentation

A 56-year-old man with end-stage renal disease on dialysis presented for evaluation of a retroperitoneal mass incidentally found on a CT scan. He was referred by his surgeon during the process of renal transplant evaluation. He reported feeling well at the time and denied a history of abdominal pain, distention, back pain, nausea, vomiting, anorexia, or change in weight. 

**Figure 1:**
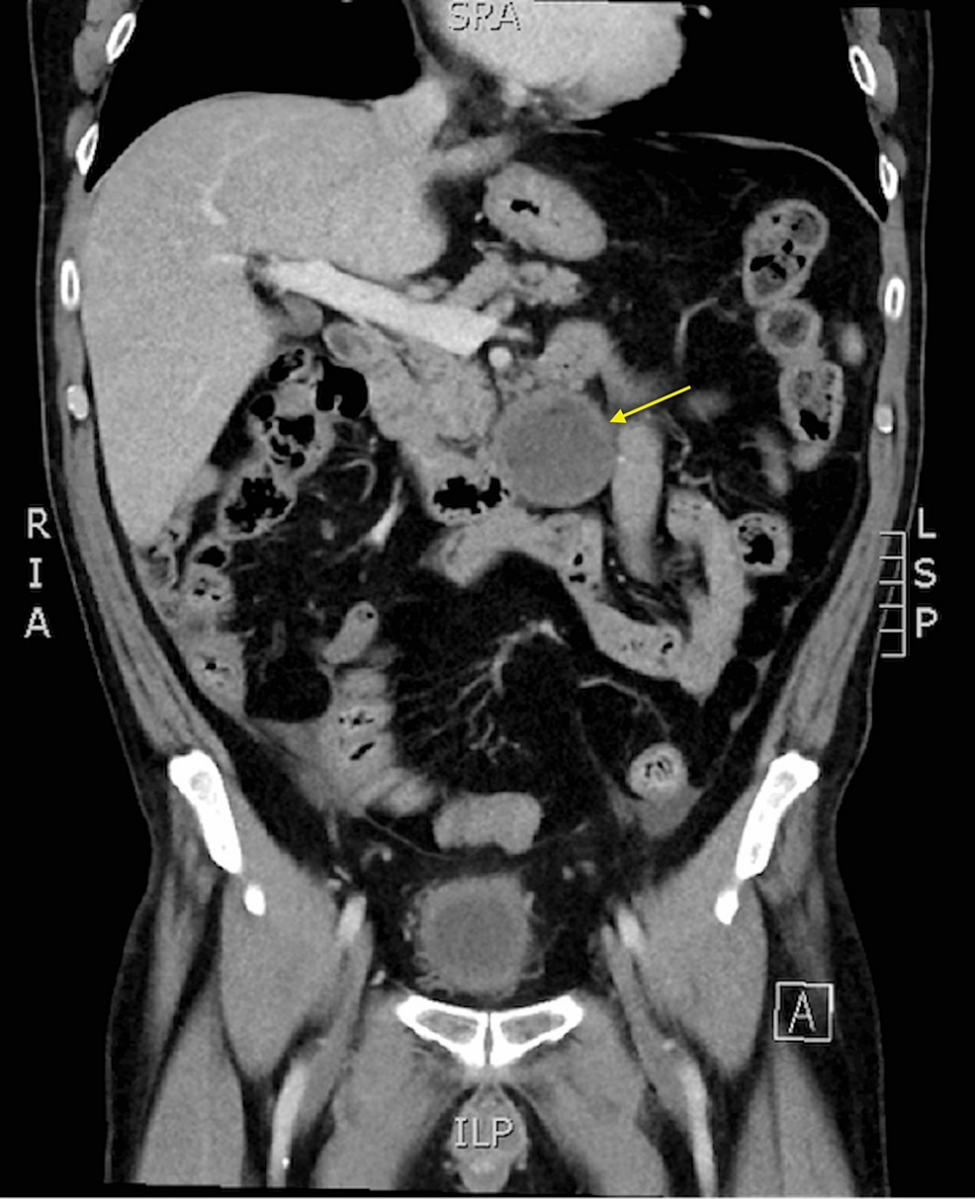
CT showing a well-circumscribed 4.9-cm round mass (arrow) abutting the fourth portion of the duodenum.

The mass was described as a 4.9-cm round lesion with central heterogeneous fat density abutting the distal fourth portion of the duodenum and extending inferiorly just left of the midline near the root of the mesentery (Figure 1). He subsequently underwent a CT-guided biopsy of the lesion, though no viable cells were recovered. Given the location of the lesion and multiple underlying comorbidities, surgical intervention was deferred in favor of endoscopic evaluation.

**Figure 2:**
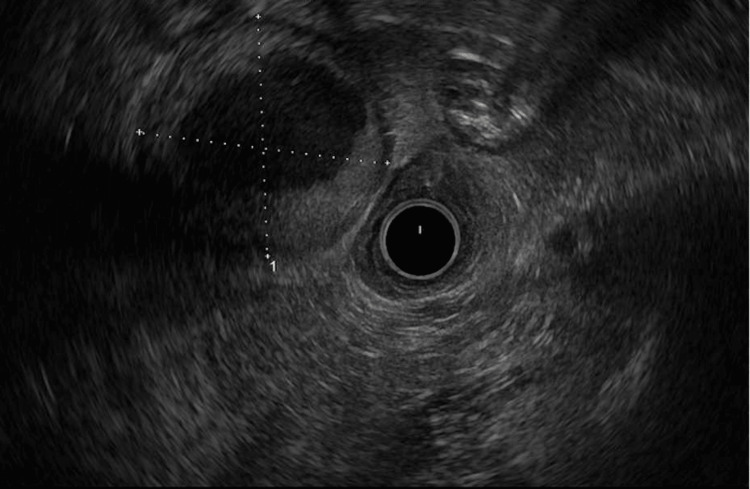
Endoscopic ultrasound delineating a 4.8 x 4.7 cm periduodenal cystic mass.

Endoscopic ultrasound (EUS)-guided biopsy/fine needle aspiration (FNA) of the lesion was performed after that. EUS revealed a 4.8 x 4.7 cm round cystic mass adjacent to the distal duodenum (Figure 2). The mass contained thick echoic and hypoechoic liquid material and appeared to abut the duodenum slightly. There was a well-formed hypoechoic rim around the lesion. No regional lymphadenopathy was noted. FNA yielded 20 mL of thin white-yellow milky fluid, which was sent for further inquiry. Core needle biopsies were also obtained. 

The fluid analysis revealed amylase 128 U/L, carcinoembryonic antigen (CEA) 4.9 ng/mL, triglycerides 41 mg/dL, and a positive qualitative chylomicron screen. Gram stain and culture analysis of the fluid were unremarkable. Pathology revealed fat necrosis and stromal fragments with chronic inflammation. No malignant cells were seen. A repeat CT scan three months later revealed trivial residual cyst fluid.

## Discussion

Mesenteric cysts are lesions that develop anywhere in the GI tract mesentery from the duodenum to the rectum, with the majority located in the small bowel mesentery [2]. This rare intra-abdominal finding is estimated to be identified in 1 in 100,000 adult hospitalizations [3]. These cysts are generally benign, with malignant transformation in less than 3% of cases [4]. Diagnosis is typically incidental as patients are largely asymptomatic though they may present with nonspecific complaints such as abdominal discomfort and distention with or without a palpable mass. Chylous mesenteric cysts, a subset of mesenteric cysts first described in 1842, represent approximately 7% of all abdominal cysts [5,6]. They occur at all ages though mostly diagnosed in infancy [7]. Their etiology is not well understood, yet these cysts are thought to arise from developmental defects in mesenteric lymphatics or following abdominal trauma [7]. Malignant risk is undefined. Due to its rarity and lack of specific symptoms, correct pre-operative diagnosis is difficult. Diagnosis is supported by ultrasound or cross-sectional imaging though definitive only by histology. 

Surgical enucleation, partial excision, drainage, and deroofing have been deemed the ideal therapeutic options up to now [1,8]. However, whenever complete excision cannot be safely performed due to adhesions of the cyst wall to surrounding mesenteric structures, resection of adjacent organs may be necessary, including bowel resection in one-third of adult cases [8]. Therefore, surgical intervention was deferred in our patient primarily due to the challenging location of the cyst, which portended a complex operation. The literature offers a few examples of EUS assessment of these cysts over the past two decades. Recognition of such asymptomatic cysts is important as clinical scenarios exist where a definitive diagnosis of the lesion is required (e.g., prior to organ transplantation). If image-guided tissue sampling is problematic or fails, diagnosis may be possible with endosonography. EUS-guided sampling may facilitate a diagnosis and avoid unnecessary surgery in cases where the cyst is adjacent to the duodenum. A return of milky aspirate should prompt evaluation for chylous mesenteric cyst via chylomicron stain.

## Conclusions

This case provides renewed data on the endoscopic evaluation of mesenteric cysts located adjacent to the duodenum. To the best of our knowledge, only one other case of EUS-guided diagnosis of a chylous cyst at this particular site has been reported. However, as this intervention is infrequent and not guideline-driven, the durability of EUS drainage and the utility of surveillance cross-sectional imaging are uncertain. Moreover, given the theoretical risk of cyst enlargement, rupture, torsion, and malignancy, it is unknown whether endoscopic drainage offers the same protection against future complications as surgical resection. For example, if the cyst wall is unable to be drained entirely, there may be a reasonable concern that cystic fluid reaccumulation will ensue. Thus, the involvement of an experienced surgeon is advantageous to comprehensive care as surgery may be merited despite endoscopy.
